# Emergency surgery in chronic intestinal pseudo-obstruction due to mitochondrial neurogastrointestinal encephalomyopathy: case reports

**DOI:** 10.1186/1755-7682-3-35

**Published:** 2010-12-08

**Authors:** Pablo Granero Castro, Sebastián Fernández Arias, María Moreno Gijón, Paloma Álvarez Martínez, José Granero Trancón, Jose Antonio Álvarez Pérez, Eduardo Lamamie Clairac, Juan José González González

**Affiliations:** 1 Department of General Surgery, Hospital Universitario Central de Asturias, Oviedo, Spain

## Abstract

Chronic intestinal pseudo-obstruction (CIPO) is a syndrome characterized by recurrent clinical episodes of intestinal obstruction in the absence of any mechanical cause occluding the gut. There are multiple causes related to this rare syndrome. Mitochondrial neurogastrointestinal encephalomyopathy (MNGIE) is one of the causes related to primary CIPO. MNGIE is caused by mutations in the gene encoding thymidine phosphorylase. These mutations lead to an accumulation of thymidine and deoxyuridine in blood and tissues of these patients. Toxic levels of these nucleosides induce mitochondrial DNA abnormalities leading to an abnormal intestinal motility.

Herein, we described two rare cases of MNGIE syndrome associated with CIPO, which needed surgical treatment for gastrointestinal complications. In one patient, intra-abdominal hypertension and compartment syndrome generated as a result of the colonic distension forced to perform emergency surgery. In the other patient, a perforated duodenal diverticulum was the cause that forced to perform surgery. There is not a definitive treatment for MNGIE syndrome and survival does not exceed 40 years of age. Surgery only should be considered in some selected patients.

## Background

Intestinal pseudo-obstruction is a rare and highly morbid syndrome characterized by impaired gastrointestinal propulsion together with symptoms and signs of bowel obstruction in the absence of any lesions occluding the gut lumen [1]. Pseudo-obstructive syndromes may be either acute (due to abdominal surgery, retroperitoneal haemorrhage, spinal or pelvic trauma, myocardial infarction, or hypokalemia) or, more commonly, chronic. The latter form, that is, chronic intestinal pseudo-obstruction (CIPO) is an important cause of chronic functional intestinal failure. CIPO can be further classified as either "secondary" to a wide array of recognized pathological conditions or "idiopathic" [2]. The diagnosis of CIPO is mainly clinical and confirmed by endoscopic or radiological exclusion of mechanical causes as well as by evidence of air-fluid levels in distended bowel loops [3]. There are multiple causes related to this rare syndrome. Mitochondrial neurogastrointestinal encephalomyopathy (MNGIE) is one of the syndromes related to CIPO. The clinical diagnosis of MNGIE disease is based on the presence of severe gastrointestinal dysmotility, cachexia, ptosis, external ophthalmoplegia, sensorimotor neuropathy, asymptomatic leukoencephalopathy as observed on brain magnetic resonance imaging (MRI), and family history consistent with autosomal recessive inheritance [4]. Direct evidence of MNGIE disease is provided by one of the following findings: increase in plasma thymidine (dThd) concentration greater than 3 μmol/l and increase in plasma deoxyuridine (dUrd) concentration greater than 5 μmol/l [5]. Thymidine phosphorylase (TP) enzyme activity in leukocytes is less than 10% of the control mean. Molecular genetic testing of *TYMP*, the gene encoding thymidine phosphorylase, detects mutations in approximately 100% of affected individuals [6]. Visceral involvement in MNGIE affects the entire gastrointestinal tract so surgery has a limited role in the treatment of these patients.

We described two rare cases of MNGIE syndrome associated with CIPO, which needed surgical treatment for gastrointestinal complications.

## Case reports

### Case 1

**Figure 1:**
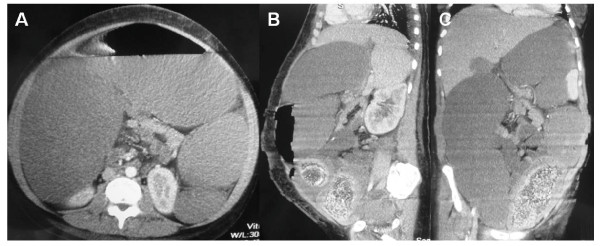
**A, B and C: Axial, sagittal and coronal abdominal CT scan images showing dilated colon**.

**Figure 2:**
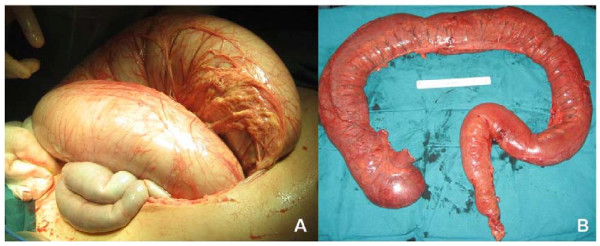
A: Intraoperative picture showing dilated colon;  B: Subtotal colectomy specimen with loss of colonic haustras.

A 27 year old woman with a history of frequent episodes of intestinal pseudo-obstruction since she was 3 years old. The patient was diagnosed in the early childhood of esophageal achalasia and urinary problems due to a lack of muscular contractility. Neurological examination revealed ptosis and external ophthalmoplegia, distal muscle weakness and lower limb hypoesthesia. Deep tendon reflexes were absent in the lower limbs. A brain MRI showed a white matter demyelinization. Laboratory tests demonstrated a low TP activity in the buffy coat associated with an increased concentration of plasma dThd (8.3 μmol/l) and dUrd (11.3 μmol/l), confirming the diagnosis of MNGIE syndrome. Clinical episodes of pain, abdominal distension and diarrhea have limited the oral intake of the patient and at the age of 18 years old she had a body mass index (BMI) of 16.2 Kg/m^2^. Oral vitamin E, coenzyme Q10, cisapride and oral erythromycin were ineffective in improving neurological and digestive symptoms. Home parenteral nutrition was started with several episodes of catheter-related sepsis that required admission. During the last hospitalization, the patient presented a new clinical episode of intestinal pseudo-obstruction that did not improve with medical treatment (nasogastric and rectal tubes, serum therapy, antibiotherapy and prokinetics). An abdominal computed tomography (CT) showed dilatation of the colon and exclusion of mechanical causes (Figure 1). Intra-abdominal hypertension and compartment syndrome generated as a result of the colonic distension forced to perform emergency surgery. A subtotal colectomy with ileostomy was performed (Figure 2). The patient was discharged in a clinically acceptable state on postoperative day 12. The histological study showed atrophy and focal fibrosis of the muscularis propria with preservation of enteric plexus.

### Case 2

A 22 year old woman with a gastrostomy since early childhood due to oral intolerance and progressive gastrointestinal dysmotility with vomiting, dysphagia, episodic abdominal pain and distension. Neurological examination revealed bilateral external ophthalmoplegia, ptosis, absent tendon reflexes and lower limb hypoesthesia. Prokinetics and oral vitamin E supplementation did not improve digestive and neurological symptoms. Diagnostic studies including a colonoscopy ruled out mechanical causes of obstruction and a diagnosis of MNGIE had been made on the basis of an increased levels of dThd (9.3 μmol/l and dUrd (12.7 μmol/l) in plasma. The patient had also been diagnosed of small bowel diverticula. Because of severe malnutrition with a BMI of 11.2 Kg/m^2^, home parenteral nutrition was started. At the age of 18 years a colectomy with ileostomy was performed due to a colonic perforation secondary to an acute episode of CIPO. Two years later, and after the diagnosis of MNGIE syndrome the patient received an allogeneic hematopoietic stem cell transplantation (HSCT) to slow down the progression of the disease with good initial response. Six months after HSCT the patient was readmitted because of a clinical episode of pain, abdominal distension and fever. An abdominal CT showed massive pneumoperitoneum due to perforated duodenal diverticulum. The patient underwent emergency surgery and a diverticulectomy was performed. Due to poor general condition and septic shock secondary to intestinal perforation, the patient died on postoperative day 12 at the intensive care unit.

## Discussion

CIPO is a rare syndrome characterized by dysfunction of gut propulsive motility which results in a clinical picture mimicking mechanical obstruction, in the absence of any mechanical process [1]. Even though CIPO is a rare syndrome, it represents up to 15% and 20% of the causes of chronic intestinal failure in children and adults, respectively [7]. Etiology may be multiple but CIPO is idiopathic in the majority of cases. Primary cases of CIPO are due to intrinsic alterations of the components of gastrointestinal wall while other cases are secondary to a variety of diseases (neurological, immune-mediated and collagen diseases, endocrine diseases) and drugs (clonidine, phenothiazines, antidepressants, antiparkinsonians, antineoplastics, bronchodilators, anthraquinones). Histopathological findings of primary CIPO include abnormalities affecting the smooth muscle cells (visceral myopathies), enteric neurones (visceral neuropathies) and interstitial cells of Cajal (mesenchymopathies) [8,9].

MNGIE is an autosomal recessive syndrome due to mutations in the TP gene and it is characterized by severe gastrointestinal dysmotility, cachexia, ptosis, ophthalmoparesis, peripheral neuropathy, white matter changes in brain magnetic resonance imaging and mitochondrial abnormalities [4]. MNGIE is the most frequent mitochondrial encephalomyopathy associated with CIPO. Deficiency of thymidine phosphorylase results in elevated concentrations of thymidine (dThd) and deoxyuridine (dUrd) in blood and tissues, as seen in our patients. Toxic levels of dThd and dUrd induce abnormalities of mitochondrial DNA (mtDNA) affecting the enzymes involved in oxidative phosphorylation. Severe depletion of mtDNA in smooth muscle cells of gastrointestinal tract and vascular wall is the most striking molecular defect of MNGIE patients [10].

Although clinical manifestations are homogeneus and recognizable, MNGIE is often misdiagnosed, particularly early in the course of the disease before all of the clinical manifestations are apparent. Gastrointestinal dysmotility is the most common presenting symptom [5]. Gastrointestinal features including dysphagia, gastroparesis, recurrent episodes of intestinal pseudo-obstruction and chronic diarrhea were confirmed in our patients. Small bowel diverticula were reported in 53% of MNGIE patients in a previous report [11]. Urinary symptoms, generally associated with evidence of urinary tract distension, are also common [9]. These two last clinical findings were seen in the cases reported here. Although myogenic involvement is more frequent, visceral abnormalities in gastrointestinal wall may be myogenic, neurogenic or both. Examination of full-thickness biopsies of the intestinal wall may help in distinguish between a myogenic or neurogenic mechanism [9,10]. Neurological signs and symptoms included peripheral polyneuropathy, leukoencephalopathy, ptosis, progressive ophtalmoplegia and hearing loss, as seen in our patients. This cluster of signs and symptoms is very specific. Other neurological symptoms are infrequent [4].

Mitochondrial dysfunction and clinical symptoms are produced after years of cumulative toxic effects of excessive nucleosides on mtDNA. Different mechanisms have been proposed to reduce circulating nucleosides as a possible therapy for MNGIE [12]. While hemodialysis to reduce nucleoside levels does not seem to be effective, platelet infusions transiently provide TP activity and reduce plasma dThd and dUrd levels [13]. Other mechanisms proposed to restore circulating TP levels are the direct administration of the stabilized active TP protein or introduction of the functional gene through viral vectors. Several drugs have been tried in the treatment of mitochondrial diseases like coenzyme Q10, vitamin K3, vitamin C or carnitine but data on their clinical efficiency are lacking [13,14]. To restore TP activity, allogeneic hematopoietic stem cell transplantation (HSCT) has been proposed as a treatment for patients with MNGIE obtaining encouraging results [15]. This therapeutic approach was performed in the case 2, with good initial clinical response. Standardization of the transplant protocol will allow evaluation of the safety and efficacy of HSCT for patients with MNGIE [16].

As seen in our patients, the clinical course of CIPO entails a progressive deterioration of bowel function and digestive symptoms. The difficulty of oral intake often leads to severe malnutrition that, in most cases required long-term parenteral nutrition. The main limitations of this nutritional support include liver insufficiency, pancreatitis, glomerulonephritis and catheter-related complications (septicemia or thrombosis). Primary CIPO due to MNGIE syndrome has a particularly poor prognosis and patients usually die around 40 years of age [17].

Pharmacological treatment of CIPO includes antiemetics, prokinetics, antispasmodics, laxatives or antidiarrheal and analgesic. Antibiotics are often useful to contrast bacterial overgrowth. As demonstrated in our patients, surgery is one of the mainstay of CIPO treatment but it has to be considered only in some carefully selected patients. A history of multiple and useless surgeries are typical of the syndrome because of the misleading digestive clinical manifestations. Benefits of surgical resections are temporally because CIPO is a progressive disease and involves the whole alimentary tract. In fact, surgery can precipitate clinical deterioration of patients. Indications for surgery must be appropriate and excessive number of surgical procedures must be avoided. Enterostomies seem to be the most logical approach in most cases. Terminal ileostomy improves digestive symptoms, decreases abdominal distension and facilitates the absorption of nutrients [9,18]. Intestinal or multivisceral transplantation should be considered when all other treatment options have failed. The complications related to this procedure such as bacterial infections are frequent and mortality rate approaches 50% at 5 years [19]. The main causes of dead in CIPO are related to parenteral nutrition, surgery, post-transplantation and septic shock of gastrointestinal origin [9].

## Conclusion

Although surgery has a limited role in the management of MNGIE patients with secondary CIPO, emergency surgery may be necessary in patients with digestive complications.

## Competing interests

The authors declare that they have no competing interests.

## Consent

Written informed consent was obtained from the patients for publication of these case reports and accompanying images. A copy of the written consents is available for review by the Editor-in-Chief of this journal.

## Authors' contributions

FSA, MMG, PAM, JGT, JAP, ELC and JGG were involved in the direct care of these patients. In addition, PGC was responsible for drafting the manuscript and JAP and JGT helped to draft the manuscript. All authors have read and approved the final manuscript.
